# The association between proportion of night shifts and musculoskeletal pain and headaches in nurses: a cross-sectional study

**DOI:** 10.1186/s12891-024-07196-5

**Published:** 2024-01-16

**Authors:** Jon Are Stavås, Kristian Bernhard Nilsen, Dagfinn Matre

**Affiliations:** 1 Faculty of Health Sciences, Oslo Metropolitan University, Oslo, Norway; 2 National Institute of Occupational Health (STAMI), Oslo, Norway; 3 Neuroscience Clinic, Department of Neurology and Department of Research and Innovation, Oslo University Hospital, Oslo, Norway

**Keywords:** Shift work, Diary questionnaire, Sleep, Mediation, Circadian rhythm, Sleep restriction

## Abstract

**Background and purpose:**

Shift work is associated with musculoskeletal pain and headaches, but little is known about how the intensity of shift work exposure is related to musculoskeletal pain and headaches. This study aimed to investigate whether a higher proportion of night shifts is associated with a higher occurrence of musculoskeletal pain and headaches. Furthermore, to investigate whether sleep duration can mediate this potential association.

**Method:**

The study included 684 nurses in rotating shift work who responded to a daily questionnaire about working hours, sleep, and pain for 28 consecutive days. The data were treated cross-sectionally.

**Results:**

A negative binomial regression analysis adjusted for age and BMI revealed that working a higher proportion of night shifts is not associated with a higher occurrence of musculoskeletal pain and headaches. On the contrary, those working ≥ 50% night shifts had a significantly lower occurrence of pain in the lower extremities than those who worked < 25% night shifts (IRR 0.69 95% CI 0.51, 0.94). There was no indication of a mediation effect with total sleep time (TST).

**Conclusion:**

The results of this study indicate that working a higher proportion of night shifts is not associated with a higher occurrence of musculoskeletal pain and headaches.

**Supplementary Information:**

The online version contains supplementary material available at 10.1186/s12891-024-07196-5.

## Introduction

An increasingly large proportion of the population is engaged in shift work [1]. To maintain a healthy workforce there is need for a better understanding of the associations between shift work and potential burdens. Shift work, particularly irregular shifts and night work, is associated with musculoskeletal pain and headaches [2, 3]. However, little is known about which shift work characteristics and “dose” that are related to musculoskeletal pain and headaches.

Musculoskeletal pain and headaches have major negative consequences for both the individual and the society [4, 5]. According to the Global Burden of Disease study, low back pain and headache disorders are the two leading causes of years of healthy life lost due to disability (YLD) worldwide [6]. Consequently, musculoskeletal pain and headaches impose vast financial and individual costs. Healthy work schedules may contribute to reduce these costs.

Musculoskeletal pain and headaches have a multifactorial origin, including work related factors, such as ergonomic, psychosocial, and organizational factors. It complicates the picture that headache may be a symptom also of e.g. temporomandibular disorders [7].

Since shift work is necessary in multiple occupational sectors and industries, it is imperative to determine which characteristics of shift work that are associated with the occurrence of musculoskeletal pain and headaches. Shift work which includes night shifts, may pose a greater health risk than shift work without nights. Longer shifts (e.g. 12-h) may pose greater risk than shorter shifts (e.g. 8-h). In a previous study, we found that night shifts was associated with increased risk of pain complaints the following day in nurses [8], and that headache tended to increase from the second to the third night shift [9]. The proportion of night shifts is a working time pattern probably relevant for health [10]. Hence, it is unclear whether a higher proportion of night shifts per unit of time is associated with musculoskeletal pain and headaches.

Reduced sleep length and disturbances in the circadian rhythm may contribute to the association between night shift and pain [11]. Poor sleep quality seems to be associated with musculoskeletal pain [12] and temporomandibular disorders [13], even in children [14]. Shift workers who work night shifts have an increased risk of experiencing reduced sleep length and sleep disturbances [15]. Reduced sleep length and sleep disturbances are associated with musculoskeletal pain and headaches [16–18]. Hence, reduced sleep length may potentially act as a mediator between night shifts and musculoskeletal pain and headaches. Potential pathomechanisms between sleep disorders and migraine may involve several sleep-related neurotransmitter systems and anatomical structures [19]. However, investigating these are outside the scope of the present study.

These knowledge gaps may be approached by investigating whether a higher proportion of night shifts is associated with a higher occurrence of musculoskeletal pain and headaches, and whether sleep duration is a part of this putative association. Filling this knowledge gap will have consequences for the design of preventive measures.

In the present study we investigate whether a higher monthly proportion of night shifts are associated with the occurrence of musculoskeletal pain and/or headaches. A few studies have investigated this association with conflicting results, and they were largely based on retrospective questionnaires with a high risk of reporting bias [20–28].

The aim of this study was to determine whether a higher proportion of night shifts is associated with a higher occurrence of musculoskeletal pain and headaches using a micro-longitudinal study design over 28 days. A secondary aim was to determine if sleep duration could mediate this association.

## Methods

### Design and study population

The data was collected in a diary study carried out by The National Institute of Occupational Health in Norway (STAMI) between October 2014 and November 2015.

An invitation was sent by post or by e-mail to a random sample of members of the Norwegian Nurses Association (*n* = 22,500). Inclusion criteria were being an authorized nurse, working in more than 50% position, being between 18 and 63 years of age, and working in a 2-shift (day, evening) or 3-shift (day, evening, night) schedule or night shifts only. Exclusion criteria were pregnancy, being breastfeeding, or having been on sick leave for more than two weeks in the last six months. We aimed for a random sample, and thus no attempts were made to balance the gender representation. A total of 5,400 participants who met the criteria received a further invitation to participate in the study. Of these, 1,032 participants answered the baseline questionnaire and, by that, consenting to participate. Of these, 727 participants also answered a diary questionnaire for up to 28 days. Of the 727 participants who responded to diary questionnaire, 43 participants were excluded due to the following reasons: they had responded less than seven days to the questionnaire (*n* = 22), not working as a nurse (*n* = 14), were on sick leave (*n* = 1), or had missing data for one of the confounding variables (*n* = 6). After correcting for the inclusion criteria, 684 nurses completed both the questionnaire at baseline at the diary questionnaire for a minimum of seven days.

### Data collection

The questionnaire at baseline contained questions about demographic conditions, lifestyle, sleep, physical and mental complaints as well as psychosocial and physical working conditions. For details on the questionnaire, see Katsifaraki et al. [29]. Participants who answered the questionnaire were included in the study. An SMS (or e-mail if not owning a smartphone) was sent to the participants daily for a total of 28 consecutive days at 21.00 o’clock with a reminder to answer a diary questionnaire as soon as possible. The diary questionnaire (details described below) asked about average pain, working hours (start and end times), and sleep. Answers received 24 h or more after the reminder, were not considered. The diary questionnaire was developed by the National Institute of Occupational Health, Norway. It was running on the smartphone’s browser and such available for both Android and IOS. Participants were assigned a unique ID, that identified their repeated responses. At the end of data collection, participants were anonymized.

### Exposure variables

Morning shift was defined as a shift that started between 05:00 and 12:00. An evening shift was defined as a shift that started between 12:01 and 18:00, while a night shift was defined as a shift that started between 18:01 and 04:59. Hence, each workday was categorized accordingly. Days without working hours were categorized as days off.

Rather than looking at associations between shift type and pain on single workdays, the present study aimed to determine whether nurses with a higher average proportion of night shifts over time, had a higher risk of reporting complaints. Therefore, the number of morning, evening, and night shifts worked for each participant for 28 days was calculated by aggregating the shift type variable. In the analysis we used proportion of night shifts, as suggested by Härmä et al. [10]. The proportion of night shifts was calculated by dividing the number of night shifts by the total number of shifts worked in the 28-day period. The proportion of night shifts was categorized as an ordinal variable. The categories used were < 25%, 25–49.9%, and ≥ 50%

### Sleep duration as a potential mediator

The diary sleep questions were derived from Carney et al. [30]. These questions were:

1. “What time did you get into bed?”, 2. “What time did you try to fall asleep?”, 3. “How long time (in hours and minutes) did it take for you to fall asleep?” which is described as sleep onset latency (SOL), 4. “How many times did you wake up, not counting your final awakening?”, 5. “In total, how long did these awakenings last?” which is described as wake after sleep onset (WASO), 6. “What time was your final awakening?”, 7. “What time did you get out of bed after your main sleep?”, 8. “Did you wake up earlier than planned?”, 9. “How long did you sleep in addition to your main sleep during the past 24 h?”.

Daily total sleep time (TST) was calculated by subtracting SOL and WASO from the time difference between when the participants was trying to sleep and the time of the final awakening. TST for all days was then summed for each participant. Furthermore, the variable was aggregated to find the number of days responded. The total sum of the TST was finally divided by the number of days responded to find the average TST. The average TST was treated as a continuous variable.

### Outcome variables

The smartphone-based diary questionnaire asked the participants to score musculoskeletal pain complaints and headache over the last 24 h on a four-point Likert scale. The categories in the questionnaire were not troubled by pain = 0, somewhat troubled by pain = 1, fairly troubled by pain = 2, and very troubled by pain = 3. Participants rated pain in five regions: the neck, shoulder, and upper back, lower back, upper extremities, lower extremities, and headaches.

In this study we wanted to study the overall effect of night shifts on pain prevalence, and not the day-to-day variation in pain intensity. Thus, for each day and pain region, the pain ratings were dichotomized and then summed for the data collection period of 28 days to find the number of days with pain. The five outcome variables were treated as discrete count variables, i.e. number of days with pain (0–28 days) from the neck, shoulder, and upper back, lower back, upper extremities, lower extremities, and headaches.

### Statistical analysis

All analyzes were done in R (RStudio Team, 2022). The estimates from the analyzes were presented with a 95% confidence interval (CI). The significance level was set at ≤ 0.05. The analysis was adjusted for age and body mass index (BMI).

**Fig. 1:**
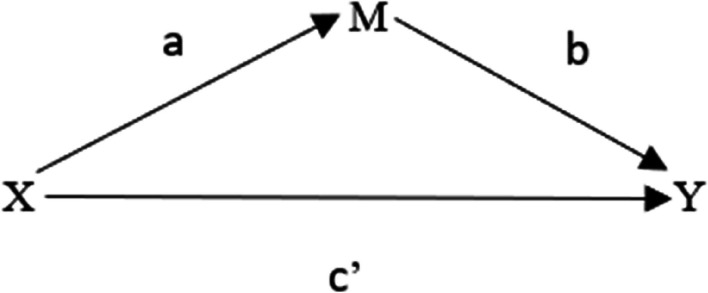
Mediation model. X is the exposure variable, M is the mediator, and Y is the outcome variable. The indirect effect is the combined effect between path a (X➜M) and path b (M ➜Y). The direct effect is path c’ (X ➜Y)

The data collected over 28 days were treated as cross-sectional data. Negative binomial regression was used, as the model was over-dispersed [31], to examine the association between the proportion of night shifts and the outcome variables. The reference category was < 25% night shifts, which was compared with 25–49.9% and ≥ 50% night shifts. Furthermore, it was investigated if TST could mediate the association between the proportion of night shifts and the outcome variables. If the combined effect for path a and path b is significant, it can be concluded that there is a mediation (see Fig. 1) [32]. The indirect effect can be found by multiplying the partial derivative of the equation for path a with respect to X by the partial derivative of the equation for path b and path c’ with respect to M [33]. Linear regression was used to analyze path a, and negative binomial regression was used for path b and path c’. As described in greater detailed in Geldhof et al. [33], when linear regression has been used in path a and negative binomial regression in path b and path c’, the conditional indirect effect can be estimated by the formula:

$${a}_{1}\times {b}_{1}{e}^{{b}_{0}+{b}_{1}\widehat{M}+{c}_{1}^{\prime}x}$$

To calculate the confidence intervals for the conditional indirect effect, bootstrap with 1000 replicates was used as proposed in Geldhof et al. [33]. In all analyses, adjustment was made for age and body mass index (BMI) as the variables theoretically can be associated with both the exposure and outcome [34–37].

In addition, an offset variable was used for all the unadjusted an adjusted analyzes with the outcome variables to adjust for the difference in number of days responded to the diary questionnaire. Offset variables can be used in negative binomial regression to adjust for differences in time for count variables [31].

## Results

### Descriptive statistics

The diary questionnaire was answered 84–85% of the days. Table 1 presents descriptive statistics consisting of demographic variables, work related variables, pain, and sleep. Mean age was 40.9 ± 11.1 years with a range of 22–63 years. Table 2 presents descriptive statistics for total sleep time (TST) for the different exposure groups used in the analysis.

**Table 1:** Descriptive statistics with demographic variables, work related variables, pain, and sleep

Variables	Participants (*n* = 684)
Sex (female), n (%)	614 (90)
Age, year, mean ± SD	40.9 ± 11.1
Body mass index, kg/m^2^, mean ± SD	24.7 ± 4.1
Percentage of full-time position (%)	
50–75%	21.6
76–90%	19.7
> 90%	58.0
Naps on the night shifts, n (%)^b^	224 (34)
Morning shift, %^c^, median (IQR)	52.9 (37.5–63.6)
Evening shift, %^c^, median (IQR)	25.5 (14.3–35.9)
Night shift, %^c^, median (IQR)	17.6 (6.7–33.3)
Neck-, shoulder-, and upper back pain, number of days, median (IQR)	4 (1–12)
Low back pain, number of days, median (IQR)	2 (0–7)
Pain in the upper extremities, number of days, median (IQR)	0 (0–3)
Pain in the lower extremities, number of days, median (IQR)	2 (0–9)
Headache, number of days, median (IQR)	4 (1–8)
TST, hours, mean ± SD	6.7 ± 1.0^a^

*SD* Standard deviation, *IQR* Interquartile range, *TST* Total sleep time

^a^*n* = 645

^b^occasionally or more often

^c^proportion of shifts in percent out of all shifts

**Table 2:** Total sleep time (TST) in hours for different proportion of night shifts

Proportion of night shifts (%)	TST, hours, mean ± SD
< 25	6.7 ± 0.9
25–49.9	6.7 ± 0.7
≥ 50	6.5 ± 1.0

### Proportion of night shifts and musculoskeletal pain and headaches

Those who worked ≥ 50% night shifts had significantly lower occurrence of pain in the lower extremities than those who worked < 25% night shifts in the adjusted analysis (IRR 0.69 95% CI 0.51, 0.94) (Table 3). For pain in other regions, no significant association was found with proportion of night shifts. As a sensitivity analysis, only nurses working a 3-shift schedule were included in the analysis. No significant associations were found in the sensitivity analysis (Table 4).

**Table 3:** Association between proportion of night shifts and musculoskeletal pain and headaches among nurses working a 2-, 3-shift schedule or night shifts only (*n* = 684). Incidence rate ratio (IRR) with 95% confidence intervals (CI) and p-values from negative b inomial regression analysis

	**Participants, n**	**Unadjusted IRR (95% CI)**	***p*-value**	**Adjusted IRR (95% CI)**^**a**^	***p*-value**
**Proportion of night shifts (%)**	**Neck-, shoulder-, and upper back pain**				
< 25	427	1.00 (ref.)		1.00 (ref.)	
25–49.9	149	0.93 (0.73, 1.17)	0.517	0.93 (0.74, 1.18)	0.566
≥ 50	108	0.86 (0.66, 1.13)	0.287	0.85 (0.65, 1.11)	0.218
**Proportion of night shifts (%)**	**Low back pain**				
< 25	427	1.00 (ref.)		1.00 (ref.)	
25–49.9	149	0.86 (0.65, 1.13)	0.279	0.89 (0.67, 1.17)	0.390
≥ 50	108	0.78 (0.57, 1.07)	0.123	0.77 (0.56, 1.05)	0.099
**Proportion of night shifts (%)**	**Pain in the upper extremities**				
< 25	427	1.00 (ref.)		1.00 (ref.)	
25–49.9	149	0.72 (0.49, 1.07)	0.107	0.79 (0.54, 1.17)	0.239
≥ 50	108	0.97 (0.62, 1.51)	0.890	0.90 (0.58, 1.39)	0.636
**Proportion of night shifts (%)**	**Pain in the lower extremities**				
< 25	427	1.00 (ref.)		1.00 (ref.)	
25–49.9	149	0.90 (0.68, 1.19)	0.458	0.93 (0.71, 1.22)	0.601
≥ 50	108	0.75 (0.55, 1.02)	0.068	**0.69 (0.51, 0.94)**	**0.019**
**Proportion of night shifts (%)**	**Headache**				
< 25	427	1.00 (ref.)		1.00 (ref.)	
25–49.9	149	1.07 (0.88, 1.29)	0.500	1.03 (0.86, 1.25)	0.729
≥ 50	108	0.98 (0.79, 1.22)	0.859	0.99 (0.80, 1.23)	0.952

^a^Adjusted for age and BMI

*p* ≤ 0,05 are in bold face

**Table 4:** Association between proportion of night shifts and musculoskeletal pain and headaches among nurses working 3-shift schedule (*n* = 551). Incidence rate ratio (IRR) with 95% confidence intervals (CI) and p-values from negative binomial regression analysis

	**Participants, n**	**Unadjusted IRR (95% CI)**	***p*-value**	**Adjusted IRR (95% CI)**^**a**^	***p*-value**
**Proportion of night shifts (%)**	**Neck-, shoulder-, and upper back pain**				
< 25	366	1.00 (ref.)		1.00 (ref.)	
25–49.9	144	0.96 (0.75, 1.23)	0.736	0.96 (0.75, 1.23)	0.741
≥ 50	41	0.97 (0.64, 1.46)	0.872	0.96 (0.64, 1.46)	0.861
**Proportion of night shifts (%)**	**Low back pain**				
< 25	366	1.00 (ref.)		1.00 (ref.)	
25–49.9	144	0.85 (0.64, 1.13)	0.265	0.87 (0.65, 1.16)	0.349
≥ 50	41	0.74 (0.46, 1.20)	0.225	0.74 (0.45, 1.19)	0.211
**Proportion of night shifts (%)**	**Pain in the upper extremities**				
< 25	366	1.00 (ref.)		1.00 (ref.)	
25–49.9	144	0.71 (0.47, 1.08)	0.111	0.77 (0.51, 1.15)	0.204
≥ 50	41	0.67 (0.33, 1.35)	0.265	0.64 (0.32, 1.27)	0.202
**Proportion of night shifts (%)**	**Pain in the lower extremities**				
< 25	366	1.00 (ref.)		1.00 (ref.)	
25–49.9	144	0.92 (0.69, 1.22)	0.571	0.94 (0.71, 1.24)	0.660
≥ 50	41	0.74 (0.46, 1.19)	0.215	0.70 (0.44, 1.13)	0.143
**Proportion of night shifts (%)**	**Headache**				
< 25	366	1.00 (ref.)		1.00 (ref.)	
25–49.9	144	1.07 (0.87, 1.30)	0.533	1.03 (0.85, 1.26)	0.747
≥ 50	41	1.02 (0.73, 1.42)	0.902	1.02 (0.73, 1.41)	0.927

^a^Adjusted for age and BMI

### Mediation analysis

No indication of a mediation effect with TST as mediator between proportion of night shifts and for any of the pain regions was found (Supplementary Table 1).

## Discussion

The aim of the present study was to determine whether the proportion of monthly night shifts was associated with the occurrence of musculoskeletal pain and headaches, and if total sleep time (TST) could mediate this potential association. The results did not support a hypothesis that a higher proportion of night shifts was associated with a higher occurrence of musculoskeletal pain and headaches. On the contrary, those working ≥ 50% night shifts had a significantly lower occurrence of pain in the lower extremities than those who worked < 25% night shifts. TST was not found to mediate the association between proportion of night shifts and musculoskeletal pain and headaches.

Previous studies that have examined the association between the proportion of night shifts and musculoskeletal pain and headaches have shown conflicting findings [20–28]. Several explanations may contribute to these inconsistencies. Firstly, the previous studies were retrospective with a potential for reporting bias [38]. Secondly, no distinction was made between those who had pain lasting only a few days and those who had pain every day over a longer period up to a year. This may have contributed to a higher number of cases classified with pain which possibly could bias the results towards the null hypothesis. Finally, the mentioned studies had different definitions for musculoskeletal pain and headaches. E.g., in the study by d’Ettorre et al. [20] work-related acute low back pain was defined as ‘activity-limiting lower back pain’ with a minimum duration of one day within 7 or 28 days. There was also a requirement that acute pain should have occurred at work. In June and Cho [21], low back pain was defined as ‘pain, aching, or stiffness’ at least once a month for one year. The different case definitions may have contributed to the different findings, depending on how strict the case definition was.

The support for a seemingly protective effect of working ≥ 50% night shifts on lower extremity pain, compared to working < 25% night shifts, could potentially be explained by lower planned activity in the hospitals during the night shift. During day/evening shifts, the nurses probably must “run faster” than during the night. In that respect, a higher proportion of night shifts imply a lower proportion of day/evening shifts, during which the nurses’ activity level is higher.

To explain why there was no significant association between the proportion of night shifts and pain in other musculoskeletal regions and headaches in this study, the so-called “healthy worker effect” may have contributed. This bias assumes that individuals with health problems, or those who are most negatively affected by the exposure, quit their jobs, or are reassigned earlier [39]. An example of “healthy worker effect” was seen in a prospective study of shift workers and the risk of being depressed, where those who were depressed at baseline had a greater risk of switching from shift work to only day work [40].

Mediation analysis with TST were carried out to better understand the mechanisms between proportion of night shifts and musculoskeletal pain and headache, despite no significant total effect was found for most of the analyses. According to Hayes and Rockwood [32], there can still be a significant indirect effect or mediation, even if there is no significant total effect. However, we did not find support for TST mediating the association between the proportion of night shifts and musculoskeletal pain and headaches. This is in contrast with a previous micro-longitudinal study based on the same data. In our previous study we found that TST could mediate the association between night shifts and lower extremity pain and abdominal pain, but this was not the case for other musculoskeletal pain and headaches [8]. The difference between the present study and our previous study by Katsifaraki et al. [8] is that in the latter study, both TST and pain were analyzed from day to day, while in the present study TST and pain were respectively analyzed as mean and number of days over several weeks. In addition, Katsifaraki et al. [8] investigated an increase in pain on a Likert-type scale from day to day, whereas in the present study pain was dichotomized each day as no pain or pain. Another study showed increased pain sensitivity the day after a night shift, but this was normalized after a night of normal sleep [41]. It is therefore conceivable that there could be an association when examining TST and an increase in pain from day to day, but not when analyzing TST and number of days with pain. Kecklund and Axelsson [11] also argued that it was uncertain whether shift workers generally experience chronic sleep loss compared to day workers. In the present study several of the participants also answered that they occasionally or more often had naps on the night shifts. This could possibly influence the association, making those working night shift less prone to musculoskeletal pain and headaches. This type of pain-reducing effect of naps during night shifts has been reported by other [42].

### Strength and limitations

Strengths of the present study are a large, randomized sample of nurses and the longitudinal data collection. The data collection takes place close to exposure and outcome. It is conceivable that this can reduce reporting bias which can often be the case with questionnaires assessing complaints over weeks or months retrospectively [38].

All analyzes were adjusted for age and BMI, as these variables can theoretically be associated with both exposure and outcome [36, 43]. The study was cross-sectional, and it was considered not relevant to adjust for baseline pain. Our research question was related to whether the participants who worked a higher proportion of night shifts had a higher occurrence of pain, and not change in pain during a night shift period. Another strength of this study is that sensitivity analysis was carried out, analyzing only the participants who worked in a 3-shifts schedule. The sensitivity analysis produced essentially similar results, confirming no association between a higher proportion of night shifts and musculoskeletal pain and headaches.

Musculoskeletal pain and headaches were measured with a four-point Likert scale, which is a type of unidimensional numeric rating scale (NRS) scale that is valid and reliable [44]. The participants had a deadline of 24 h to answer the diary questionnaire. Additionally, in this study, the cut-off value for pain was set to a minimum of “somewhat troubled”, and the variable was categorized as no pain or pain. Therefore, this study only distinguished between no pain and pain each day, making it difficult to compare the prevalence of pain to similar studies with a retrospective design. However, it is a strength that the sample was based on a randomized drawing from the Norwegian Nurses Association and 90% of the participant was women, which is similar to the nurse population in Norway [45], supporting the generalizability of this study, although the results will be more representative for females than men.

A diary is preferable to a retrospective questionnaire, as sleep can vary greatly from night to night. Diaries have also been considered the gold standard for subjective sleep reporting [46]. It is recommended to answer sleep diary questionnaire within one hour after the end of a sleep period [30]. However, in the present study subjects were asked to report pain and sleep the last 24 h. The sleep measures in this study may therefore have been prone to bias. It is also a limitation that we asked about exposure (work start and end times) and outcome (pain) in the same questionnaire.

A limitation of this study is that the number of days data was collected from the diary questionnaire varied between seven and 28 days among the participants. Although the diary data collection lasted 28 days, the number of participants who completed the questionnaire became fewer towards the end of the period. Generally, excluding participants with missing data may increase the risk of selection bias, and including them may increase the risk of information bias. To adjust for different number of days reported in the diary, an offset variable was used, which was the natural logarithm of the number of days answered on the diary questionnaire. This has also been done in other studies to adjust for time for follow-up [47]. Thus, it is the rate of days with pain and not the number of days with pain that is analyzed [31]. Another limitation of this study is that shift length (e.g. 8-h vs. 12-h schedules) was not taken into account. Also, a limitation of the study is that the diary questionnaire did not allow us to specify types of headaches. Finally, it is a limitation that the genetic predispositions to sleep problems and to musculoskeletal disorders was not assessed.

## Concluding remarks

The results indicate that working many night shifts is not associated with a higher occurrence of musculoskeletal pain and headache. If replicated, the results may have important implications for the design of healthy work schedules, indicating that factors other than the proportion of night shifts are important for the association between shift work and complaints of musculoskeletal pain and headache.

### Supplementary Information

**Additional file 1:** **Supplementary Table 1.** Estimates for the conditional direct effects and the conditional indirect effects for total sleep time (TST) as a potential mediator for the association between proportion of night shifts and musculoskeletal pain and headaches among nurses (*n*=645). Presented with incidence rate ratio (IRR) with 95 % confidence intervals (CI).

## Data Availability

No datasets were generated or analysed during the current study.

## Abbreviations

BMI: Body mass index
NRS: Numeric rating scale
STAMI: The National Institute of Occupational Health in Norway
TST: Total sleep time
YLD: Years of healthy life lost due to disability
